# Intersection of Epigenetic and Metabolic Regulation of Histone Modifications in Acute Myeloid Leukemia

**DOI:** 10.3389/fonc.2019.00432

**Published:** 2019-05-22

**Authors:** Abhinav Dhall, Barry M. Zee, Fangxue Yan, M. Andres Blanco

**Affiliations:** ^1^Newborn Medicine, Boston Children's Hospital, Boston, MA, United States; ^2^Department of Biomedical Sciences, School of Veterinary Medicine, University of Pennsylvania, Philadelphia, PA, United States

**Keywords:** AML—acute myeloid leukaemia, metabolism, histone methlyation, HDACs, epigenetics (methylation/demethylation)

## Abstract

Acute myeloid leukemia (AML) is one of the most lethal blood cancers, accounting for close to a quarter of a million annual deaths worldwide. Even though genetically heterogeneous, all AMLs are characterized by two interrelated features—blocked differentiation and high proliferative capacity. Despite significant progress in our understanding of the molecular and genetic basis of AML, the treatment of AMLs with chemotherapeutic regimens has remained largely unchanged in the past 30 years. In this review, we will consider the role of two cellular processes, metabolism and epigenetics, in the development and progression of AML and highlight the studies that suggest an interconnection of therapeutic importance between the two. Large-scale whole-exome sequencing of AML patients has revealed the presence of mutations, translocations or duplications in several epigenetic effectors such as DNMT3, MLL, ASXL1, and TET2, often times co-occuring with mutations in metabolic enzymes such as IDH1 and IDH2. These mutations often result in impaired enzymatic activity which leads to an altered epigenetic landscape through dysregulation of chromatin modifications such as DNA methylation, histone acetylation and methylation. We will discuss the role of enzymes that are responsible for establishing these modifications, namely histone acetyl transferases (HAT), histone methyl transferases (HMT), demethylases (KDMs), and deacetylases (HDAC), and also highlight the merits and demerits of using inhibitors that target these enzymes. Furthermore, we will tie in the metabolic regulation of co-factors such as acetyl-CoA, SAM, and α-ketoglutarate that are utilized by these enzymes and examine the role of metabolic inhibitors as a treatment option for AML. In doing so, we hope to stimulate interest in this topic and help generate a rationale for the consideration of the combinatorial use of metabolic and epigenetic inhibitors for the treatment of AML.

## Introduction

All cancers are a result of highly proliferative cells but accompanying blockade in differentiation is particularly pronounced in certain leukemias such as AML. The process of differentiation typically induces two fundamental cellular programs: halting of proliferation and acquisition of a permanent cellular identity. The former entails dramatic metabolic state changes, and the latter invokes chromatin-based epigenetic dynamics. Notably, the enzymology of mitochondrial and chromatin reactions is well-appreciated to be biochemically related, potentially enabling their dual-regulation. Accordingly, we hypothesize that pro-differentiation therapeutic strategies—even in non-APL AMLs—may be highly effective if both metabolic and chromatin pathways are targeted simultaneously. To date, differentiation therapy with all *trans-*retinoic acid (ATRA) and arsenic trioxide (ATO) has been curative in the promyelocytic AML subtype (1), which provides a promising precedence.

We will highlight the epigenetic and metabolic pathways that are dysregulated in AML and also affect chromatin modifications which are critical for orchestrasting differentiation and locking in the differentiated cellular state. Furthermore, we will discuss the numerous clinical trials that have evaluated the efficacy of metabolic and epigenetic inhibitors and also highlight the combinatorial use of these inhibitors which has provided some promising results.

## Metabolic Rewiring in AML

Rapidly proliferating tumor cells exert a vastly different nutrient demand on their microenvironment in comparison to the surrounding benign tissue. Metabolic rewiring in cancer cells is largely responsible for generating this distinct nutrient demand and has been a topic of extensive research for the past few decades (2). One of the first landmark discoveries of oncogenic metabolic dysregulation was made during the 1920s when Otto Warburg observed increased glycolysis by tumor cells even under normoxic conditions (3). This switch in glucose metabolism from primarily mitochondrial tricarboxylic acid cycle to cytosolic anaerobic glycolysis allows cancer cells to maintain low levels of reactive oxygen species (ROS) while also ensuring an adequate supply of biomolecules to facilitate tumor growth (4, 5). In the blood, these programs are more complicated. In both AML and their normal hematopoietic counterparts, metabolic states are dynamic, in part due to the hierarchical cell identity programs that characterize each one. Healthy hematopoietic stem cells (HSCs), which reside in the typically oxygen-poor bone marrow, are usually quiescent and non-proliferative. These features are consistent with their observed anaerobic glycolysis and low production of reactive oxygen species (ROS) (6). However, upon commitment to differentiation, these cells enter the cell cycle and transition to oxidative phosphorylation. Interestingly, this situation is reversed in AML; the lowly proliferative leukemia stem cells (LSCs) respire, whereas highly proliferative AML blast elevate glycolysis (7). Notably, altered metabolism is one of the strikingly few differences between HSCs and disease-sustaining LSCs, suggesting its importance in AML cell identity (8). This unique metabolic feature of LSCs may be of significant therapeutic relevance. Inhibition of Bcl-2 impedes oxidative phosphorylation of LSCs and selectively eliminates this population in AML(9). While the Bcl-2 inhibitor Venetoclax is currently being tested as a single agent in clinical trials (10), recent studies have also shown that Venetoclax in combination with DNMT inhibitor Azacytidine has more striking effects in targeting LSCs in AML patients (11–13). This synergistic effect implies that metabolic and epigenetic programs are intertwined in AML. This intriguing relationship, and the possibility of combination targeting of the two, warrants further exploration of this rising area.

## Epigenetic Reprogramming in AML

Epigenetic programs are similarly awry in AML compared to normal hematopoiesis. In AML, epigenetic modifiers are strongly overrepresented among recurrently mutated driver genes, and mounting evidence suggests their critical role in enforcing the differentiation arrested cell fate. Biochemical evidence suggests that the altered metabolic and epigenetic programs of AML may not be independent of one another. A number of biomolecules produced during glucose metabolism are further utilized as cofactors by enzymes that act as “epigenetic effectors” and regulate the expression of numerous tumor suppressors and oncogenes (Table 1) (14). The best studied example of metabolic dysregulation that directly alters the chromatin state in a subset of AMLs are mutations in the isocitrate dehydrogenase (IDH1 and IDH2) enzymes. Excitingly, IDH1/2 inhibitors have shown great promise in reversing the differentiation block and improving patient prognosis (15–17). However, we propose that the mitochondria-chromatin connection can be exploited in cases beyond IDH1/2 mutant AMLs. We derive this view in part from models of normal myeloid cell differentiation programs. Numerous studies have demonstrated the interaction between metabolic dynamics and epigenome regulation in classical cell fate models such as macrophage activation and trained immunity [reviewed in (18)] Upon classical macrophage activation by LPS stimulation, a switch from oxidative phosphorylation to glycolysis occurs rapidly, producing a buildup of lactate—which has been shown to directly antagonize class II HDACs (19). In contrast, in trained immunity models of beta-glucan-treated monocytes, glutaminolysis induced fumurate buildup, leading to inhibition of KDM5 family demethylases and sustained H3K4me3 levels at open chromatin (20). We propose that programs such as these—perhaps in altered or misfunctional form—are also directly relevant to AML biology, as differentiation blockade and cell fate dysregulation are hallmarks of this disease. Below, we will discuss current literature that highlights pathways that have shown promise therapeutically along with new pathways that could be targeted for future development of therapeutics against AML.

**Table 1:** Metabolic enzymes involved in the generation of metabolites/cofactors utilized by important epigenetic effectors.

**Metabolite/Cofactor**	**Metabolic enzymes**			**Epigenetic enzymes**
	**Mitochondrial**	**Cytosolic**	**Nuclear**	**Nuclear**
Acetyl-CoA	ACSS1, PDC	ACSS2	ACLY, ACSS2	KAT
SAM	–	MAT	MAT	KMT, PRMT
FAD	FADS	FADS	FADS	LSD1/LSD2
Alpha-ketoglutarate	IDH1-3, GLUD1,2	IDH1,2	–	JmjC demethylases, TET1-3
NAD	NMNAT3	NMNAT2, NADSYN	NMNAT1	Sirtuins, PARP

*ACLY, ATP citrate lyase; ACSS, Acetyl-CoA Synthetase short-chain family member; FADS, Flavin Adenine Dinucleotide Synthetase; GLUD, Glutamate dehydrogenase; IDH, Isocitrate Dehydrogenase; KAT, Lysine acetyl transferase; KMT, Lysine methyl transferase; LSD, Lysine specific demethylase; MAT, Methionine Adenosyltransferase; NADSYN, NAD synthetase; NMNAT, Nicotinamide mononucleotide adenylyltransferase; PARP, Poly ADP ribose polymerase; PDC, Pyruvate dehydrogenase complex; PRMT, Protein arginine methyl transferase*.

## Histone Acetylation

Histone acetylation is an enzymatically regulated post-translational modification (PTM), under the control of at least 17 acetyltransferases (HATs) that require an acetyl-CoA cofactor and of at least 18 deacetylases (HDACs) that require an NAD^+^ or zinc cofactor (21). Several lines of evidence suggest that the activities of HATs and HDACs are coupled with the overall metabolic state of a cell. Higher rate of glycolysis in pluripotent cells relative to differentiated lineages accounts for both increased availability of acetyl-CoA and increased histone acetylation (22). On the other hand, non-APL AML blasts generally have reduced acetylation of histone H3 (23) and histone H4 compared to normal white blood cells (24).

## Epigenetic Dysregulation of Histone Acetylation

Some of the most dramatic examples of impaired acetylation regulation involve chromosomal rearrangements that fuse HAT enzymes to other genes. Multiple rare AML subsets contain genetic fusions with HAT partners (25). For instance, the chimeric transcript of t(11;16)(q23;p13.3), t(8;16)(p11;p13), and t(8;22)(p11;q13) AMLs codes for MLL-CREBBP, MOZ-CREBBP, and MOZ-EP300 fusions, respectively. Interestingly, the MOZ-CREBBP fusion is a more potent activator of NF-κB-dependent promoters than either wild type protein alone (26) and synergizes with hyperactivated FLT3 to promote NF-kB target transcription. The relevance of HATs to hematological malignancies is not limited to cases where the enzymes are fusion partners. The proliferation of MLL-AF9 and NUP98-HOXA9 driven leukemia depends on the acetyltransferase activity of MOF, such that MOF mutants incapable of binding acetyl-CoA via a domain deletion or amino acid substitution have impaired colony formation efficiency (27). However, the relevance of HATs in AML can extend beyond acetyltransferase activity. For example, dissociation of the protein interaction between Myb and EP300 with Celastrol acts independently with inhibition of EP300 acetyltransferase activity with respect to repressing Myb-activated gene targets (28). Accordingly, the combined effect of inhibiting both EP300 enzymatic activity as well its interaction with Myb has been shown to induce synergistic myeloid differentiation in *in vitro* models. These studies highlight the importance of and distinction between the enzymatic activities and the scaffolding functions of HATs in promoting AML.

**Figure 1:**
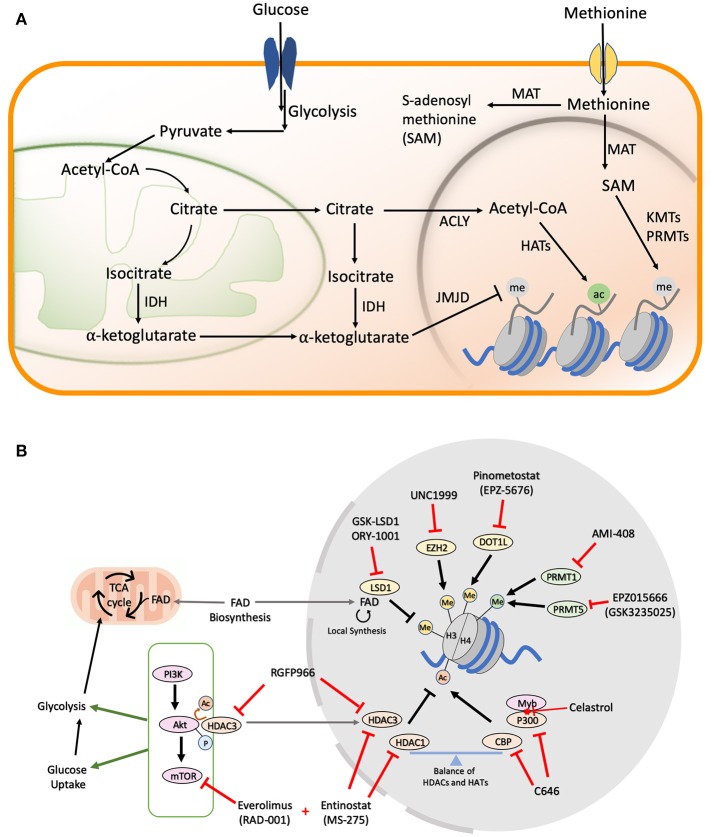
**(A)** Metabolic pathways in cells. Majority of the cofactors utilized by epigenetic effectors are produced as a result of glucose and methionine metabolism. The metabolic products can be transported from the mitochondria or cytoplasm to the nucleus where they help catalyze various histone modifications. **(B)** Drug Targets in AML Metabolic and Epigenetic Pathways. A schematic figure shows examples of drug inhibitors that target metabolic and/or epigenetic regulators in AML. Me, Methylation; Ac, Acetylation; P, Phosphorylation; FAD, Flavin Adenine Dinucleotide.

Disruption of histone acetylation homeostasis may have context-specific effects in AML. Decreasing histone acetylation with the HAT inhibitor C646 (Figure 1B), which preferentially targets the catalytic activity of CREBBP and the related acetyltransferase EP300 (also known as KAT3B), significantly reduced the proliferation of 8 out of 10 tested AML tissue culture cell lines (29). Surprisingly, however, increasing histone acetylation with the HDAC inhibitor valproic acid yields similar effects, potently inducing differentiation of AML1/ETO-driven Kasumi-1 cells. Notably, the equivalent dose of valproic acid does not significantly impair the proliferation of normal murine hematopoietic lin^−^ progenitors (30). These different findings suggest the balance of HAT and HDAC activity, rather than total acetylation prevalence, is a critical component of AML malignancy. While poor selectivity and bioavailability of HAT inhibitors has prevented their advance to the clinic (31), HDAC inhibitors (HDACi) have shown therapeutic efficacy. The FDA has recently approved multiple HDAC inhibitors for the treatment of T cell lymphomas and multiple myeloma, including the pan-HDACis Vorinostat, Panobinostat and Belinostat, and the class I specific HDACi Romidepsin. Several studies have evaluated the efficacy of HDACi in AML with promising results (32). In an *in vivo* model of t(8;21) driven AML, administration of Panobinostat triggered proteasomal degradation of the pathogenic AML1/ETO fusion protein leading to terminal myeloid differentiation and excellent survival benefit in mice (33). Unfortunately, the activity of Panobinostat was limited to this subset of AML, prompting the search for novel combinatorial therapies. However, phase I/II studies using combination of Panobinostat [10–40 mg orally, thrice weekly for seven doses (34) or 20 mg orally for six doses (35)] with Azacitidine failed to report significant improvement in the overall survival of patients and was also associated with severe Panobinostat related side effects of fatigue, nausea, syncope and somnolence. The toxicities associated with pan-HDAC inhibition warrant the need to develop more selective HDACi with improved benefit–risk profiles (36).

## Metabolic Regulation of Histone Acetylation

Unlike phosphorylation, which is impervious to changes in cellular levels of ATP due to the overabundance of this small molecule, the levels of histone acetylation are intricately linked to the nuclear pool of acetyl-CoA (Figure 1A) (37), which is largely maintained by the ATP citrate lyase (ACLY) enzyme that utilizes glucose-derived citrate as its substrate (38). The PI3K/Akt/mTOR pathway is a master regulator of cellular glucose intake (39), and also activates ACLY to maintain acetyl-coA in the nucleus under nutrient starvation conditions (40). Multiple cancers exploit this pathway to confer growth advantage under limited nutrition conditions, making the PI3K/Akt/mTOR axis an attractive therapeutic target (41). Constitutive activation of PI3K/Akt/mTOR signaling is observed in >60% of AML patients and is also correlated with aggressive disease progression, as well as shorter overall survival times (42, 43). Rapamycin and its analogs (rapalogs), which were initially approved as immunosuppressive drugs to prevent organ transplant rejection, have found significant use as inhibitors of the mTORC1 complex in AML. Initial *in vitro* studies demonstrated promising cytostatic effects upon rapamycin administration in K562, U937, HEL, HL60, KG1, and KG1a AML cell lines (44). However, moderate clinical effects have been observed with rapalogs alone due to the complex interplay between PI3K, Akt, and mTOR proteins leading to rapid development of drug resistance (43, 45). To overcome these limitations, simultaneous inhibition of multiple pathways in the PI3K/Akt/mTOR axis has been the focus of next generation of dual inhibitors (46). Dactolisib or NVP-BEZ235 is a dual inhibitor of the PI3K and mTOR signaling which was shown to block the clonogenic ability and proliferation of multiple primary AML cells and cell lines with low nM doses (47). Along with establishing the safety and efficacy of the new dual inhibitors, multiple phase I/II trails are also ongoing to evaluate the efficacy of combining rapalogs with chemotherapeutic agents (48, 49).

Even though single drug therapies have so far been the major focus in the attempt to restore the balance of histone acetylation in secondary and refractory AML, the most promising therapeutic leads have come from the simultaneous inhibition of HDACs and the mTOR/Akt axis. Entinostat, which primarily inhibits HDAC 1 and 3, partially inhibits Akt/mTOR signaling in HL60 AML cells. However, combination of Entinostat with the mTOR inhibitor Everolimus yields excellent synergism in inducing differentiation and apoptosis in myeloblasts (50). More recently, HDAC3 was found to directly interact with and regulate the Akt protein in leukemic cells. Accordingly, the HDAC3-specific inhibitor, RGFP966 (Figure 1B), which sensitizes MLL/AF9 AMLs to Doxocycline and Cytarabine, could also be useful in preventing drug resistance, as it suppresses Akt activation (51). The study fell short of combining HDAC and mTOR/Akt inhibitors in the long term to understand the mechanism of drug resistance development, but similar findings in other cancers point toward a general mechanism of overcoming drug resistance associated with the combination of HDACi and mTOR inhibitors (52).

## Histone Methylation

**Figure 2:**
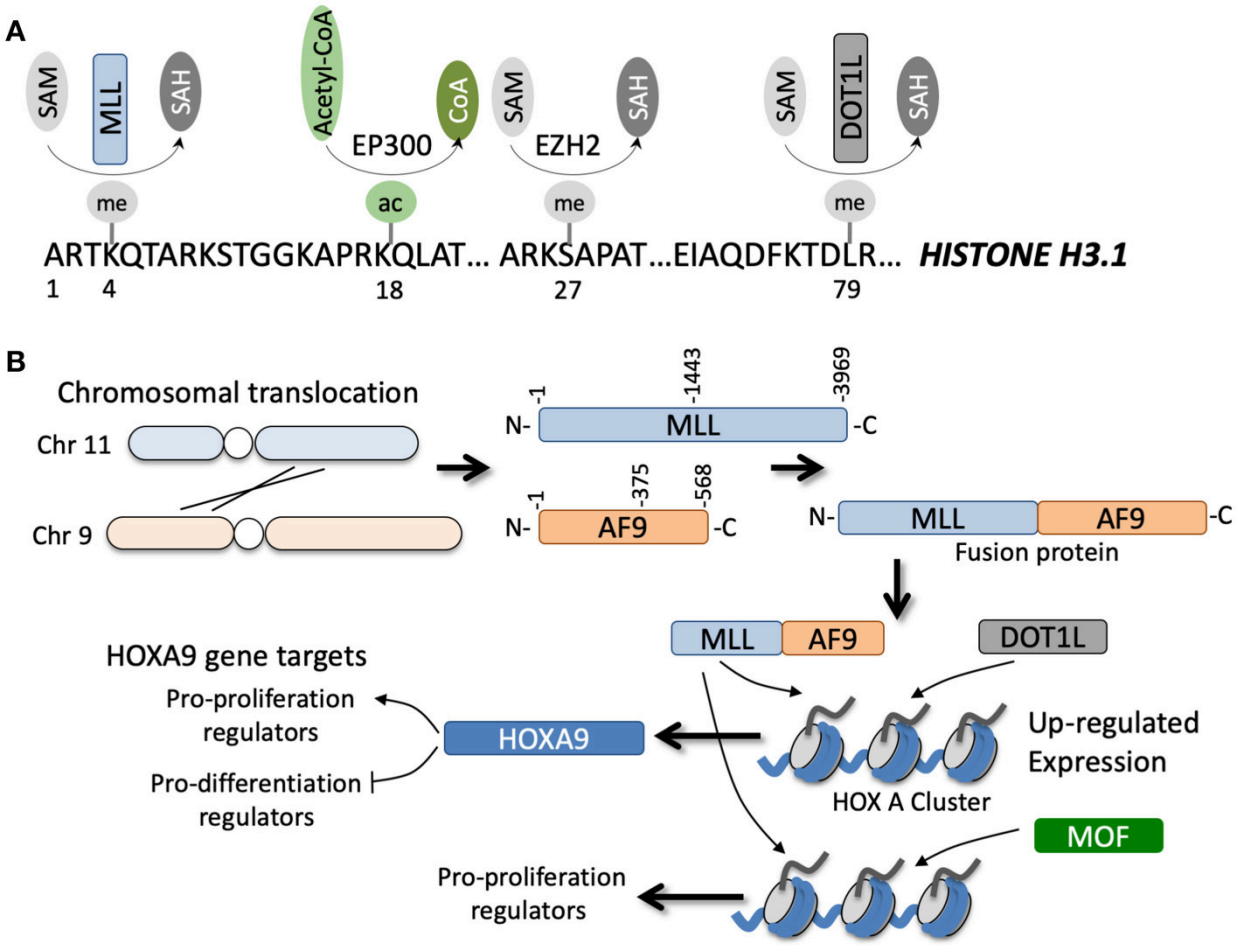
**(A)** Specific amino acids along the N-terminal region of histone H3.1 can be post-translationally modified by various enzymes in a co-factor dependent manner. **(B)** Example of a chromosomal translocation event between chromosomes 11 and 9 that leads to gene fusion of MLL and AF9. The fusion occurs at various junctions, but generally leads to transcription and translation of an MLL-AF9 fusion protein (note the reciprocal AF9-MLL protein can also be generated but is not shown). The fusion protein can coordinate with DOT1L methyltransferase or MOF acetyltransferase to epigenetically regulate numerous gene targets, for example genes in the HOXA cluster, that directly and indirectly promote tumorigenesis.

Post-translational histone methylation of lysine and arginine residues is well-documented and arguably more complex in regulation and function than histone acetylation (Figure 2A). While a lysine can only acquire a single acetyl group, it can acquire up to three methyl groups, and an arginine can acquire one or two methyl groups arranged in symmetric (i.e., one methyl group per side chain amine) or asymmetric (i.e., both methyl groups on the same side chain amine) manner (53). Furthermore, unlike histone acetylation, which is generally associated with active gene expression, histone methylation is associated with either active or repressed gene expression, depending on the site and degree of methylation (54). The methyl donor used by all histone methyltransferases (HMTs) is S-adenosyl methionine (SAM), which is derived chiefly from the condensation of methionine and ATP, and is one of the links between the metabolic state of a cell and its epigenetic landscape (55).

## Epigenetic Dysregulation of Histone Methylation

Of all the known HMTs, KMT2A or the MLL methyltransferase has the most well-documented role in the development of acute leukemia. MLL translocations are observed in ~10% of all AML cases (Figure 2B) and are often associated with poor prognosis (56). To date, 135 different MLL rearrangements have been identified in acute leukemias, but almost 90% of the MLL rearrangements observed in AML patients are with the translocation partners AF6/9/10, ELL, ENL, and SEPT6 (57). Downstream upregulation of HOXA9 and MEIS1 is a common feature of a large majority of these MLL fusions and is essential for driving leukemogenesis (58). Adding to this complexity, the regulation of the Hox cluster of genes in MLL fusion driven leukemias is strongly associated with H3K79 methylation, a mark associated with transcriptional elongation, rather than the canonical MLL substrate H3K4 (59). Hence, inhibition of Dot1L, the only known H3K79 methyltransferase in humans, is a promising therapeutic strategy against AML, although recently concluded phase I studies have shown modest effects of a highly specific Dot1L inhibitor, Pinometostat (EPZ-5676) (60).

The H3K4 mono and di-methylation specific, FAD-dependent histone demethylase LSD1 (also known as KDM1A) has also been found to be crucial for both normal hematopoiesis and AML malignancy (61). While LSD1 is ubiquitously expressed in all tissues, up to 32% of CML patients and 56% of myelodysplastic syndrome (MDS) patients have elevated levels of the protein in bone marrow biopsies (62). Additional genome-wide knockdown screens have revealed that growth of AML cell lines, relative to other non-AML cancer types, are especially sensitive to LSD1 depletion (63). In support for the central role of LSD1 in AML, pharmacological inhibition of LSD1 with GSK-LSD1 and ORY-1001, both of which irreversibly inactivate the enzyme, leads to significant reduction in proliferation of various AML tissue culture lines and increase in survival of murine disease models (64, 65). However, the essential nature of LSD1 in ensuring normal differentiation of hematopoietic progenitors (66) and the ability of tranylcypromine (Parnate) based LSD1 inhibitors to cross the blood brain barrier results in severe side effects when used in high doses as a monotherapy. New generation of reversible inhibitors that selectively target LSD1's interaction with other proteins such as CoREST could show therapeutic benefits without the neurological side effects (67). Alternatively, combination of low-dose LSD1 inhibition with inhibitors of metabolic pathways may provide another avenue to induce therapeutic differentiation in myeloblasts.

Other methylation regulators, in particular the methyltransferase EZH2, which is a subunit of the Polycomb repressive complex 2 (PRC2), is frequently mutated in myeloid malignancies. EZH2 is responsible for di- and tri-methylation of Lys27 in H3 to establish a repressive chromatin state at promoters of important hematopoietic transcription factors such as HOXA9 (68). Although loss-of-function mutations in EZH2 primarily result in myelodysplastic/myeloproliferative neoplasms (MDS/MPN) (69), the catalytic activity of EZH2 was also shown to be essential for rapid AML progression (70). Hence, inhibition of EZH2 catalysis has been explored as a therapeutic strategy in AML, first with pan methyltransferase inhibitors such as 3-Deazaneplanocin A (DZNep) in combination with the HDAC inhibitor Panobinostat (71), and more recently with specific EZH1/2 inhibitors such as OR-S1/S2 (72) and UNC1999 (73). Unfortunately, many EZH2 inhibitors fail to show robust effects *in vivo* as a monotherapy compared to the current standard of care chemotherapy, prompting the combination of multiple epigenetic inhibitors as a treatment option for AML (74). However, this work needs to proceed with caution, as EZH2 has also been reported to help prevent development of resistance against multiple drugs (75).

The various methylation states of arginine residues in histones adds additional complexity to the histone methylome. Three classes of arginine methyltransferases (PRMTs) have been identified in mammals so far. Class I generates omega-N^G^, N^′G^-asymmetric dimethylarginine (ADMA), Class II catalyzes omega-N^G^, N^′G^-symmetric dimethylarginine (SDMA), and Class III stops at the production of omega-N^G^-monomethylarginine (MMA). The first identified arginine methyltransferase, PRMT1, has been shown to directly associate with a variety of oncogenic fusion proteins. Shia et al. demonstrated that PRMT1 interaction with RUNX1 fusion proteins mediates transcriptional activation through increasing promoter H4R3me2a of multiple genes regulating proliferation (76). Subsequently, Cheung et al. found that several MLL and non-MLL fusion proteins, such as MOZ-TIF2, require interaction with PRMT1 and other epigenetic modifiers such as the H3K9ac demethylase KDM4C to drive their oncogenic transcriptional programs (77). The H4R3 residue plays additional roles in leukemogenesis through its symmetric dimethylation by PRMT5. PRMT5 has been suggested to influence the transcription of critical genes in AML. Tarighat et al. found that PRMT5 activates the *FLT3* gene and suppresses miR-29b, resulting in increased proliferation of myeloblasts (78). Intriguingly, chemical inhibition of PRMT5 using EPZ015666 (GSK3235025) partially reversed the differentiation blockade of *MLL-*rearranged AMLs and slowed disease progression *in vivo* (79). The safety, pharmacokinetics and pharmacodynamics of these newly developed PRMT5 inhibitors, including EPZ015666, are currently being investigated in clinical trials and hold promise as an alternate line of attack against various cancers.

## Metabolic Regulation of Histone Methylation

Similar to acetylation, small changes in the cellular levels of SAM can differentially affect the activity of various histone methyl transferases (80, 81). Human ESCs/iPSCs, which are known to rely on glycolysis for their energy production needs, have been shown to depend on high levels of SAM to maintain pluripotency and proliferation (82). Although global dynamics of metabolism-responsive histone lysine and arginine methylation have not been examined, Lys4 in H3 displays a strong association with metabolism. In an elegant study by Mentch et al., a direct and reversible relationship of H3K4me3 with methionine depletion was observed in human cell lines and in mice. SAM depletion under methionine restriction induced loss of promoter H3K4me3 at oncogenes such as AKT1 and MYC, leading to their downregulation. However, feedback to one-carbon metabolism pathways eventually allowed for partial restoration of H3K4me3 at several promoters (83). This compensation is most likely achieved through decreased consumption of SAM by alternate pathways and by downregulation of certain H3K4 demethylases. Consequently, this suggests that, in order to effectively modulate H3K4me3 levels at promoters, a combination of metabolic regulation of SAM and inhibition of H3K4 specific HMTs or demethylases is required. Indeed, a domain focused CRISPR screen has recently shown that knocking out the catalytic domains of certain HMTs and histone demethylases has profound effect on the survival of murine MLL-AF9/Nras^G12D^ AML cells (54).

Lysines demethylases are equally adept at responding to the metabolic state of a cell and are known to rely on two types of cofactors; either flavin adenine dinucleotide (FAD) in case of LSD1/LSD2, or 2-oxoglutarate (α-ketoglutarate), oxygen and iron Fe(II) in case of the Jumanji domain containing demethylases (84). The role of α-ketoglutarate in relation to leukemogenesis is perhaps the most well-studied example of metabolic dysregulation and has been extensively discussed in several reviews (85, 86). Additionally, many JmjC-containing histone demethylases (JARID1B, JMJD1A, JMJD2B, and JMJD2C) show increased expression under hypoxic metabolic conditions as a result of direct HIF-1 promoter binding to compensate for their decreased catalytic activity under limited O_2_ conditions (87). A more direct role of KDM4A in sensing cellular oxygen was recently proposed when its catalytic activity demonstrated a graded response to hypoxia in U2OS cells (88). On the other hand, prolonged hypoxia had no effect on the protein levels of LSD1, but reduced its catalytic activity through a gradual decrease in cellular FAD levels (89). This finding makes sense, as O_2_ is essential for the reoxidation of FADH_2_ back to FAD after a round of catalysis by LSD1. However, the presence of more than 100 flavoproteins inside a cell makes it difficult to isolate the epigenetics-related effects of FAD metabolism in cancer development. Nonetheless, new reports challenging the long held view of FAD synthesis being restricted to mitochondria and cytosol have opened the possibility that nuclear pools of FAD can have localized effects on the demethylation activity of LDS1/LSD2 (90). How these effects play out in the hypoxic microenvironment of the bone marrow (0.5–4% O_2_) still needs to be investigated, because clues from these metabolic studies will provide new and exciting pathways that can be targeted in conjunction with well-studied epigenetic pathways as future therapeutics in leukemias.

## Discussion

Thus far, we have reviewed evidence demonstrating how metabolic states can directly influence chromatin-based epigenetic programs in AML. The shared biochemistry of mitochondrial and chromatin regulation is common to all eukaryotic cells. However, the rapid tissue turnover in the blood requires orchestration of rapidly changing cell identity programs, creating unique mitochondrial dynamics. From the quiescence of HSCs to the proliferative bursting of progenitors to cell cycle exit upon terminal differentiation, blood cells can phase through markedly different epigenetic and metabolic states in a short period of time. However, to become leukemic, these cells must not only elevate proliferative potential, but must also disable differentiation programs. We propose the possibility that altered metabolic dynamics may help simultaneously confer both of these features upon AML cells, as metabolic alterations not only enable the rapid proliferation of blasts, but also directly skew the epigenetic programs that regulate differentiation. This scenario would be distinct from solid tumors, in which the metabolic switch to aerobic glycolysis is primarily thought to enable rapid proliferation. Indeed, it is possible that metabolic alterations in fact represent a very key mechanism through which the AML differentiation block is constructed.

One prediction of this hypothesis is that experimental manipulation of cellular metabolic programs in normal blood cells *in vivo* may impair their differentiation programs in a manner that resembles the AML differentiation block. There is support for this prediction. For example, a mouse knockout study investigated the blood phenotypes resulting from loss of the mitochondrial phosphatase PTPMT1, which is highly expressed in HSCs (91). Hematopoietic loss of PTPMT1 hampered oxidative phosphorylation, elevated glycolysis and decreased oxygen consumption. Most intriguingly, blood-specific ablation of PTPMT1 markedly arrested myeloid differentiation programs and induced a large buildup of HSCs and phenotypic multipotent progenitors (MPPs). Additionally, in an elegant study that isolated the stage of maturation from myeloid progenitors to granulocytes, Lee et al. (92) found that inhibition of the key nutrient sensor mTORC1 blocked terminal differentiation and allowed continuous growth of GMPs *in vivo*. Such studies demonstrate that metabolic alterations in healthy myeloid cells can induce an AML-like differentiation block. It will be of interest to see how exactly the corresponding alterations in chromatin regulators may contribute to the gene expression program driving the observed differentiation arrest.

Finally, the connection between metabolism and chromatin regulation in AML may have therapeutic significance. Epigenetic regulators have emerged as attractive therapeutic targets for AML, and we have highlighted several inhibitors of chromatin factors that have shown potential in AML models. However, as with most cancer therapeutics, these drugs may work better in combination than as monotherapies. We propose that it would be of particular interest to explore AML drug combinations that pair an epigenetic inhibitor with a metabolic inhibitor. Our rationale is that metabolic inhibitors will have profound influences on chromatin regulators, perhaps opening up marked epigenetic vulnerabilities. For example, if a metabolic inhibitor decreases the pool of nuclear acetyl-CoA, cells may struggle to maintain proper histone acetylation levels—which in turn could make them exquisitely vulnerable to HAT inhibitors, as any further impairment of histone acetylation could be intolerable. The number of possible combinations between metabolic and chromatin regulators is large, but advances in screening methodology—both genetic and small molecule—is starting to make such endeavors feasible.

## Author Contributions

AD and AB conceptualized the idea and wrote majority of the manuscript. BZ and FY helped write parts of the manuscript and prepared the figures.

### Conflict of Interest Statement

The authors declare that the research was conducted in the absence of any commercial or financial relationships that could be construed as a potential conflict of interest.
